# Efficacy of an inhibitor of GSK‐3β in an Alzheimer’s Disease Mouse Model

**DOI:** 10.1002/alz.093368

**Published:** 2025-01-03

**Authors:** Mobeena Ghuman, Allal Boutajangout, Abdurrahman Atta, Rim El Kaddioui, Alan P Kozikowski, Thomas Wisniewski

**Affiliations:** ^1^ Center for Cognitive Neurology, New York, NY USA; ^2^ NYU Grossman School of Medicine, New York, NY USA; ^3^ Actuate Therapeutics Inc, Illinois, IL USA; ^4^ Center for Cognitive Neurology, New York University Langone Health, New York, NY USA

## Abstract

**Background:**

Neurofibrillary tangles (NFTs), one of the hallmarks of Alzheimer’s disease (AD), are composed of highly phosphorylated forms of the microtubule‐associated protein tau. Phosphorylation results from the activity of several threonine/serine kinases, and increased expression of glycogen synthase kinase‐3β (GSK‐3β). These are involved in the formation of paired helical filament (PHF)‐tau, which induces the formation of NFTs. Preclinical and clinical studies have supported that GSK‐3β inhibition may have therapeutic benefits for AD. We assessed the therapeutic effect of our novel GSK‐3β inhibitor, ING‐135, in an animal model of AD.

**Method:**

hTau/PS1 mouse models were injected intraperitoneally with ING‐135 (30 mg/Kg; n = 15 per group) starting at 3 months of age, three times a week for three months, and control mice received saline only. Animals went through different behavioral tasks and brain tissue was subsequently harvested for analysis of treatment efficacy.

**Result:**

Treated animals (n = 15) did not show significant differences when compared to controls on sensorimotor tasks (i.e., Traverse beam, Rotarod, and Locomotor activity). Significant behavioral improvement was noted in treated mice on three tasks using a closed field symmetrical maze (Day 1 one‐tailed t‐test p = 0.0036; Day 2 one‐tailed t‐test p = 0.0118; Day 3 one‐tailed t‐test p = 0.0002). Immunohistochemistry analysis was performed on brain sections of treated animals and controls, using monoclonal antibodies PHF1 and CP13. Histology analysis showed a reduction of tau pathology in the motor cortex and hippocampus of treated animals (one‐tailed t‐test p = 0.0001, p = 0.0002; respectively). No significant differences were observed between the two groups with GFAP, and Iba‐1 showed a reduction of microglia in the cortical area (p = 0.05). Results were not significantly different in the dentate gyrus. Further biochemical analyses are underway.

**Conclusion:**

Our novel inhibitor of GSK‐3β (ING‐135), improves cognitive decline (long‐term memory) and decreases tau phosphorylation in treated animals versus controls, in the absence of toxicity.

